# The return of the Iberian lynx to Portugal: local voices

**DOI:** 10.1186/s13002-017-0200-9

**Published:** 2018-01-11

**Authors:** Margarida Lopes-Fernandes, Clara Espírito-Santo, Amélia Frazão-Moreira

**Affiliations:** 1 Divisão de Conservação da Biodiversidade, Instituto da Conservação da Natureza e Florestas (ICNF), Avenida da República, 16, 1069-040 Lisboa, Portugal; 2 Faculdade de Ciências Sociais e Humanas/Nova, Centro em Rede de Investigação em Antropologia (CRIA-FCSH/NOVA), Avenida de Berna, 26, 1069-061 Lisboa, Portugal; 3 Grupo Lobo, Departamento de Biologia Animal, Faculdade de Ciências da Universidade de Lisboa, C2, Campo Grande, 1749-016 Lisboa, Portugal; 4 Montes de Encanto, Rua da Charnequinha, 5, Parracheira, 2420-026 Arrabal, Portugal

**Keywords:** Ethnoecology, Iberian lynx, Perceptions, Portuguese protected areas, Reintroduction

## Abstract

**Background:**

Ethnographic research can help to establish dialog between conservationists and local people in reintroduction areas. Considering that predator reintroductions may cause local resistance, we assessed attitudes of different key actor profiles to the return of the Iberian lynx (*Lynx pardinus*) to Portugal before reintroduction started in 2015. We aimed to characterize a social context from an ethnoecological perspective, including factors such as local knowledge, perceptions, emotions, and opinions.

**Methods:**

We conducted semi-structured interviews (*n* = 131) in three different protected areas and observed practices and public meetings in order to describe reintroduction contestation, emotional involvement with the species, and local perceptions about conservation. Detailed content data analysis was undertaken and an open-ended codification of citations was performed with the support of ATLAS.ti. Besides the qualitative analyses, we further explored statistic associations between knowledge and opinions and compared different geographical areas and hunters with non-hunters among key actors.

**Results:**

Local ecological knowledge encompassed the lynx but was not shared by the whole community. Both similarities and differences between local and scientific knowledge about the lynx were found. The discrepancies with scientific findings were not necessarily a predictor of negative attitudes towards reintroduction. Contestation issues around reintroduction differ between geographical areas but did not hinder an emotional attachment to the species and its identification as a territory emblem. Among local voices, financial compensation was significantly associated to hunters and nature tourism was cited the most frequent advantage of lynx presence. Materialistic discourses existed in parallel with non-economic factors and the existence of moral agreement with its protection.

The considerable criticism and reference to restrictions by local actors concerning protected areas and conservation projects indicated the experience of an imposed model of nature conservation. Opinions about participation in the reintroduction process highlighted the need for a closer dialog between all actors and administration.

**Conclusions:**

Local voices analyzed through an ethnoecological perspective provide several views on reintroduction and nature conservation. They follow two main global trends of environmental discourse: (1) nature becomes a commodified object to exploit while contestation about wildlife is centered on financial return and (2) emblematic wild species create an emotional attachment, become symbolic, and gather moral agreement for nature protection.

Lynx reintroduction has been not only just a nature protection theme but also a negotiation process with administration. Western rural communities are not the “noble savages” and nature protectors as are other traditional groups, and actors tend to claim for benefits in a situation of reintroduction. Both parties comprehend a similar version of appropriated nature.

Understanding complexity and diverse interests in local communities are useful in not oversimplifying local positions towards predator conservation. We recommend that professional conservation teams rethink their image among local populations and increase proximity with different types of key actors.

## Background

**Fig. 1:**
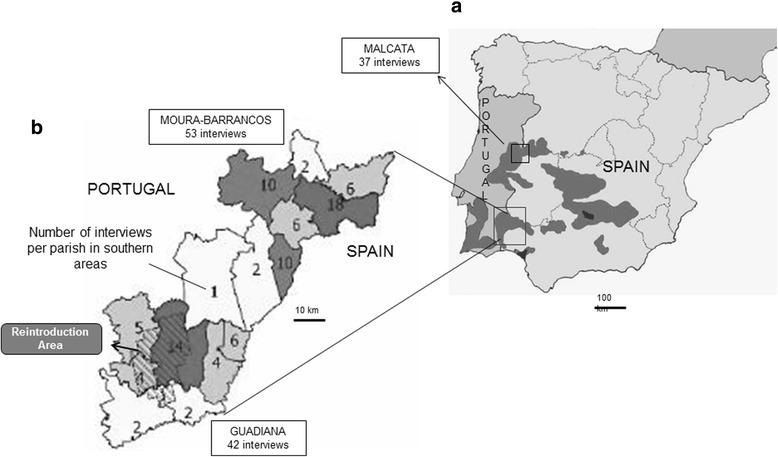
Study areas. **a** Iberian lynx distribution in 1990s (gray) and 2010s (black) with study area denoted. Adapted from Ward [79] with data from Delibes et al. [2] and Guzmán et al. [3]. **b** Sampling for social study in reintroduction area. Distribution of interviews in the study area

The Iberian lynx is one of the last large carnivores coexisting with humans in Europe, sharing many attributes in ecological terms with the Wolf or the Eurasian Lynx. It is a top predator endemic to the Iberian Peninsula facing a high risk of extinction, presently classified as endangered, mainly due to regression of its main prey––the wild rabbit [1]. This species was once distributed throughout southeastern Iberia, but towards the end of the twentieth century, it was restricted to two remnant and depleted populations in the south of Spain [2–4] (Fig. 1). Following extinction of small populations, there was a steady range contraction and no successful colonization of other areas [5]. A captive breeding program started in the early 2000s with the goal of creating viable specimens for reintroduction of the species into the wild [6]. In Portugal, the last vestiges of the species’ former presence date back to 1997 and 2001 [7, 8], but breeding populations were no longer thought to be present [9]. Although not formally extinct, lynx presence was considered no longer recoverable in Portuguese historical areas without a reintroduction program.

Reintroductions of wild species are complex processes, but they have much public appeal and draw a high level of attention from the media [10]. They might represent, in fact, a major human response to the species extinction crisis of the Anthropocene era [11]. A recent overview of 72 reintroduction projects worldwide presents a growing percentage of biological success (58% were successful or highly successful), but there are cases of documented failures [12]. A reasonable number concerns mammals, exemplified in Europe by the case of the Eurasian lynx which started in the 1970s. One of the main challenges identified in this last project concerned human dimensions, namely conflicts with hunters and weak political will [13].

Iberian lynx reintroduction recently started on a broader scale in Iberia. In Portugal, 27 animals were released between 2015 and 2017 in the southern Guadiana area (ICNF data). Supported by a captive breeding program, the reintroduction plan foresees coexistence with humans, once more, in several locations of Iberia following extinction in most of its former range (LIFE project Iberlince NAT/ES/000570). However, the recent emergence of a new variant hemorrhagic disease [14] caused a decline in wild rabbit abundance and brought uncertainty to the process as well as possible social contestation over the return of a predator.

The Iberian lynx is a wild rabbit specialist [15–17], and a female lynx needs a high wild rabbit abundance to establish a territory of around 5 km^2^ [18]. Lynxes act as superpredators with respect to other carnivores, i.e., they expel foxes, mongooses, and other medium-sized carnivores from their territory [19]. Although biological aspects of the species are well known, studies about the relationship of humans and Iberian lynx are scarce, focused on economic conflict [20], and only recently has the social visibility of the species been explored with an anthropologic gaze [21].

Reintroductions present important opportunities for multidisciplinary studies and, among social sciences, an interesting ethnoecological context in which close interactions between people and wildlife take place. Social and ecological issues might meet to characterize a particular momentum. The role of Social Sciences in conservation science and practice is more and more recognized nowadays within an interdisciplinary approach in which Ethnobiology and its specific methodologies play a distinctive role [22]. Assessing local knowledge has become an essential task of the discipline and grassroots research seeks to understand people interpreting external information such as conservation of biodiversity [23]. Documenting knowledge, perceptions, and concerns about species and conservation projects provides valuable information for decision makers. It also offers the opportunity for collaborative decision-making which might be more effective than top-down decisions. Understanding underlying biodiversity conflicts and promoting trust in stakeholders increases the likelihood of positive biodiversity outcomes [24]. The present study was integrated in an Iberian conservation project (LIFE Iberlince) and part of a wider ethnographic research from which we present here part of the data collected. We aim to characterize a scenario from an ethnoecological perspective as defined above in which dialog between conservationists and local people can be established.

Some studies on attitudes about wildlife yielded a considerable amount of information concerning conservation of threatened species (e.g., [25]) and in particular large carnivores (e.g., [26–33]), but generally, those surveys tend to focus either on support or opposition from the public. Several studies have considered the human dimensions of wild species reintroductions emphasizing conflicts or simply measuring acceptance among the general public (e.g., [34–36]).

Concerning Iberian lynx, a telephonic survey was conducted in pre-selected areas for lynx reintroduction in Andalusia (Spain) and found a remarkably high social support for the project [37]. In Portugal, data on attitudes towards the lynx and the black vulture were collected together [38], and the survey suggested positivity as well as considerable ambivalence towards general lynx protection. An anthropological approach was therefore opportune which included specific questions about reintroduction and focused on local key actors. Such an approach has been used to study knowledge and perceptions about carnivores [39–41], but we are unaware of any anthropological studies about carnivore reintroductions. Anthropology has analytical tools providing insights vital to the success of conservation efforts [42], and it can also have an important role for governance in protected areas [43]. An *emic* point of view from locals can unveil the underlying relationship between humans and non-humans and social issues that need to be addressed.

Our aim in this study was to understand the attitudes of local key actors towards the lynx and towards the process of its reintroduction. We assumed attitude to be the result of several factors such as knowledge, beliefs, values, emotions, opinions, and experiences. Attitude is part of what characterizes a culture, and all of those factors can influence an intention towards lynx presence. Thus, we consider the disposition towards a situation or an object, which Bourdieu [44] includes in *habitus* definition. We were also interested in the variations between geographical areas and different key actors. Understanding attitudes with a multidimensional in-depth approach aimed to explain reasons for the resistance sometimes found to carnivore return to territories (e.g., [45]). We gathered further information about the relationship of local actors with conservation initiatives and with the establishment of the protected area. Overall, we aimed to build an ethnoecological portrait of humans and predators in a rural Western context.

## Methods

We conducted a non-random sample of semi-structured interviews choosing key actors for lynx conservation in areas pre-selected for reintroduction by the administration based on existing biological variables. The aim was to get a representative sample among people with the interests and capacity for decision-making over the appropriate sub-areas for lynx reintroduction. These key actors are not necessarily representative of all locals but are particularly significant for conservation either by having the capacity to change land management (land owners including livestock breeders), regulating or promoting new activities (technicians), representing administrative counties (council representatives), or by developing new interests and uses in the area (nature recreation). These actors were previously identified and contacted to be interviewed rather than randomly selected. A balanced number of each type of actor was approached as well as an equivalent number of hunters and non-hunters (Table 1). We also sought people who had direct contact with the species––lynx observers (mainly in the past)––and followed a “snow ball” methodology [46] to get privileged contacts among local wildlife “specialists.” Category saturation during data collection was reached and taken as an indicator of good coverage of opinions and range of perceptions.

**Table 1:** Number of interviews per key actor and geographical area

Key actor	Moura-Barrancos	Guadiana	Malcata	Total
Land owners (including livestock breeders)	9	9	5	23
Local council representatives	8	8	8	24
Local nature conservation technicians (administration, NGOs, fiscalization)	6	7	7	20
Land and hunting managers	6	8	2	16
Hunting guards	8	3	3	14
Nature activity users and promoters (tourism, collectors, leisure)	6	7	5	18
Lynx observers	9	-	7	16
Total	52	42	37	*131*
Hunter	26	19	15	60
Non-hunter	26	23	22	71

Key actors were residents in two adjacent potential lynx areas in southern Portugal––Moura-Barrancos and Guadiana, which was the site of eventual reintroduction. An additional historical lynx area in central Portugal––Malcata––was later sampled. This last area has long been associated with the species. Sampling different areas allowed us to compare attitudes and enlarge the total number of key actors interviewed.

The interview lasted approximately 1 h and followed an outline of 35 open questions. We used unlabeled image cards of carnivores to address local memory and practices with carnivores; knowledge about the lynx’s diet, territory size, and superpredator effect; personal will to observe a lynx in the wild, opinion about reintroduction, and about environmental institutions (see “Appendix” section). During a period of 90 days between 2012 and 2014, a total of 94 semi-structured interviews in both southern areas of Moura-Barrancos (MB) and Guadiana (G) were conducted mainly by the first author (*n* = 88). An ethnographic approach [47] was followed in these areas, which included observation and participation in some local practices, such as hunts, hunting management routine, olive harvesting, and livestock guarding. Informal conversations and local public meetings on natural resources management with administration or NGOs were registered. Later, in 2015, there was an opportunity to extend the research to Malcata (M) where a total of 37 interviews were conducted by the second author.

Available key actors with the profiles previously selected were mostly men (with the exception of ten women in MB/G and four in M) aged between 31 and 80. Education level varied from primary school to post graduate studies.

In the reintroduction area (G) and Moura-Barrancos, interviews were geographically distributed throughout all local councils (Fig. 1); in Malcata area, key actors were mostly concentrated in one council (Penamacor) where the Natural Reserve is located.

Interviews were recorded (if authorized) and later transcribed. A detailed content data analysis was done, and an open-ended codification of citations was performed with the support of ATLAS.ti. Besides the qualitative analyses, we did complementary quantitative analysis to further explore results and possible associations between variables. From the answers obtained concerning lynx future presence with reintroduction, we developed an opinion scale which varied between − 1 to + 2 according to being: unfavorable (− 1), indifferent (0), favorable with conditions (+ 1), and favorable without conditions (+ 2).

The biological knowledge was also divided into categories for analytical purposes. In the case of carnivores, previous studies indicated that knowledge of a species and favorable attitudes towards their conservation were associated (e.g., [26, 48, 49]). In order to statistically test that association in our case study and compare results, it was necessary to compare local knowledge and scientific knowledge. We constructed scales whereby a higher score indicated a closer match to scientific literature: (a) diet––does not know or answers “meat” (0), wild rabbit and other prey (+ 1), wild rabbit specialist or mainly rabbit (+2); b) territory––does not know or 25–100 km^2^ or > 100 km^2^ (0), 5–25 km^2^ (+ 1), and ≤ 5 km^2”^ (+ 2); (c) superpredator effect––thinks that lynx has no effect or that carnivores are stronger (− 1), does not know (0), has doubts (+ 1), admits that lynx might have an effect (+ 2), knows that lynx has a superpredator effect (+ 3).

Significant differences between attitude variables were generally tested using chi-square, and respective *p* values are presented in the results accordingly. Geographical areas were compared using a Kruskal-Wallis. Correlation between knowledge and reintroduction opinion scales was tested using Spearman’s coefficient. Statistical analyses were carried out using IBM SPSS statistics software (version 20).

### Study areas–the local context

The history of land management in southern Portugal is characterized by the existence of large private estates with multi-agro exploitation including cereals, cattle, and cork oak forest. Most residents were rural workers; poverty levels were high and education levels low. A political revolution in the country in 1974 changed the social scenario; cereal production decreased but the primary sector kept being the most important for local economy. Agriculture was mechanized but a considerable proportion of the territory still belongs to a few land owners and is extensively used. Although income and life conditions improved in the last decades, these inland areas suffered much emigration or rural exodus and population decrease. Land management options have been strongly conditioned by EU agriculture policies and subsequent subsidies. Nowadays, the region presents areas of scrubland, sheep pasture lands, and pine afforestation. All territory is used privately or by hunting associations, and there exist local exploitation practices of natural products such as honey and mushrooms.

The Natural Park of Guadiana was created in 1993, and Natura 2000 site of Moura-Barrancos was classified in 1997 since when further human activities are limited by administration authorizations. No direct “removal of people” or “economic displacement” took place, as for instance Brockington and Igoe [50] describe. LIFE conservation projects have been conducted in these areas since 2006 by NGOs and administration (http://habitatlinceabutre.lpn.pt/; http://www.iberlince.eu). A diverse fauna which includes several threatened predators attract birdwatchers, and tourism has been increasing. Red foxes (*Vulpes vulpes*) and Egyptian mongooses (*Herpestes ichneumon*) are common species in the area, and it is legal to hunt them.

Malcata is an inland area in central Portugal where human population density is similarly low due to emigration. Unemployment is high, industry is scarce, and nature tourism is still in its early stages. Most properties are smaller than 5 ha, and olive trees are the exception to a decrease in all kinds of agricultural crops. Agriculture is based on livestock use in permanent pastures. Pine trees and eucalyptus have been exploited since intense forestation took place in the 1970s, which significantly changed local natural habitats and land use.

Malcata has a Nature Reserve, created in 1981 after a public petition to save the Iberian lynx and its habitat. The area is dominated by heathland with some typical Mediterranean species. Hunting, mainly big game, has been a traditional activity only practiced outside the natural reserve, and there are few farming or agricultural practices here.

## Results

### Welcomed and contested reintroduction

The opinions of the key actors about reintroduction are summarized in Table 2. Most interviewees were fairly positive about the possibility of lynxes living in their region, particularly in Malcata. The highest percentage of negative positions from key actors was in Guadiana, the actual reintroduction area.

**Table 2:** Percentage of opinions among informants about lynx reintroduction in Guadiana, Malcata, and Moura-Barrancos

Opinion about reintroduction	Positive (%)	Ambivalence (%)	Negative (%)	Indifference or lack of opinion (%)	Indicates locations for reintroduction (%)
Guadiana	52	14	17	14	33
Moura-Barrancos	67	8	9	10	45
Malcata	89	0	8	3	92

The same was true for ambivalence and indifference or lack of opinion, which were higher in Guadiana.

Resistance was justified by informants mainly with the arguments of scarcity of wild rabbit for both predators and hunting, which has an important economic value. There was a general narrative of competition between hunters and carnivores which made lynx unwelcome for certain actors:

I do not think it will have conditions because (…) it will damage hunting and that type of economic activity has some importance (…) and if it is a protected species there will be problems straight away. (Council representative, G, 2014)

The positive positions and acceptance were, in turn, conditioned to certain factors described freely by the informants. Those conditions constituted the main local contestation, and we organized them into the categories as presented on Table 3 (number of occurrences indicated).

**Table 3:** Conditions for lynx presence indicated by interviewees (*n*) in the three geographical areas

Conditions concerning reintroduction	*n*	Guadiana	Moura-Barrancos	Malcata	Key actors
Higher wild rabbit abundance	55	√	√	√^a^	Mainly land owners and local council representatives
Local awareness campaigns	26	√^a^	√	√	All key actors
Financial compensation	22	√^a^	√		Not mentioned by lynx observers or nature activity users Hunters^a^
Reestablishment of agricultural practices for wild rabbit abundance	20	√	√	√^a^	
More suitable habitat for lynxes	16	√	√	√	
Agreements with proprietors and hunters	15	√	√	√	
No restrictions (to hunting and others)	15	√	√	√	Not mentioned by land owners
Marketing of lynx as a tourist attraction	4	√	√		
No hunting in lynx areas	4		√	√	
Social acceptance	3	√	√		
Hunting fee reduction	2	√			

Absence of a tick signifies that the condition was not mentioned in the respective area

^a^The condition was mentioned significantly more in that area (*p* < 0.05)

Most of these conditions, referred to in all areas, were related to human exploitation of rabbit and again with rivalry with predators. “Financial compensation” was a condition mentioned significantly more in Guadiana and by hunters when compared with non-hunters (*p* = 0.046). There was a clear perception of hunters’ contestation voices in Guadiana, and the need for local awareness campaigns to be directed at them was raised. Furthermore, the possibility that the lynx could be killed was spontaneously mentioned in 41% of interviews in both Guadiana and Moura-Barrancos together (*n* = 39). Key actors consider some areas “unsafe” and mentioned the existence of threats for lynx such as predator control and illegal practices like poisoning. This reference to the possibility of lynx being killed, in Guadiana, seems also to have been a “warning message” to decision makers prior to lynx releases. It was a display of locals’ potential power of action in situ towards a wild species in response to a unilateral decision.

### Local knowledge and scientific literature

Nearly all interviewees identified lynx by photograph, showing that the species was already well recognized locally. Local ecological knowledge included the lynx, in particular in Moura-Barrancos. Diverse aspects about lynx ecology were mentioned by interviewees (*n* = 18) such as being a solitary animal, secretive, elusive, needing tranquility, hunting near rabbit burrows, and having been a scarce species. Lynx habitat was also known by interviewees (*n* = 23) and mainly defined as scrubland with further references to the presence of water, trees, open areas to hunt and rocky areas used as dens.

It is really a lively animal, beautiful and - it jumps like mad. And when they hunt, they hunt from a great distance! They go, even on stubble, putting their paws down… we don’t hear a thing! They jump the height of a wall, with the prey in their mouth. (Lynx observer, MB 2013)

It is a cat, larger. The lynx, at least from the experience we have, used to take its meals next to the rabbits, in the burrows (…). Once we figured that one entrance was bigger (…) we got to know it when the little ones came out (reference to cubs) (…). (Manager, MB 2013)

It wouldn’t cause harm (lynx presence) - they eat one or two rabbits a day: wild beasts eat little, it is the instinct of nature, to eat little. (Lynx observer, MB 2013)

This knowledge tends to be exclusive to residents, often hunters, who have spent much time in the terrain, identified signs of wildlife presence, participated in night expeditions, or captured carnivores in traps. They are local wildlife “specialists” with a particular/specialized knowledge that is not common or shared by the whole community. Nowadays, in these rural areas, lynx and other wild species are more commonly known indirectly through media. We verified that television was an important means by which wildlife is presently known rather than direct experience. Interviewees refer to it as their source of personal knowledge about the lynx (*n* = 23).

As the lynx would be, at the time of interview, a new occurrence, or a returnee to one of the areas, we also assessed specific knowledge of key actors about the species to explore associations with opinions about reintroduction.

**Fig. 2:**
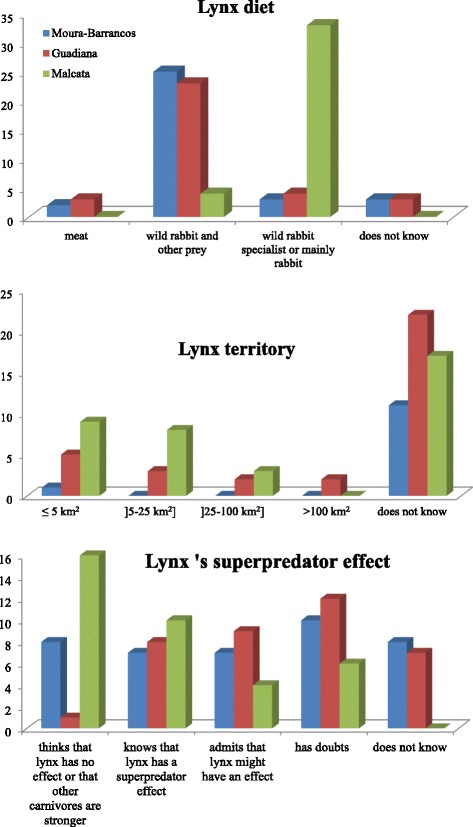
Knowledge about lynx’s diet, territory, and superpredator effect in the three different areas of study. In each graph, the categories more similar to scientific knowledge were “wild rabbit specialist or mainly rabbit” (diet), “≤ 5 km^2^” and “5–25 km^2^” (territory), and “knows that lynx has a superpredator effect” (superpredator effect)

Figure 2 summarizes actors’ knowledge about three aspects of lynx biology which were considered relevant in a reintroduction scenario––diet, territory, and superpredator effect––as the predator could be seen as a competitor for game and for space. A common concern expressed in interviews in Guadiana and Moura-Barrancos was that the lynx was a competitor for hunting (*n* = 22).

Categories presented in graphs were built from open-ended responses and the number of respondents counted. Concerning lynx diet, most informants associated Iberian lynx with wild rabbit. This knowledge coincides with the scientific knowledge from diet studies, which all indicate the “specialist” character of a stable lynx population consuming more than 85% of this type of prey only [15–17]. Fewer respondents considered that the species diet was almost exclusively wild rabbit. This specialization can be important to understand the threat status of Iberian lynx and not to expect a great impact from a resident lynx population on livestock. In Moura-Barrancos and Guadiana, 34% of informants specifically referred to livestock consumption as part of the lynx diet. It is documented that Iberian lynx occasionally consumes livestock and preys on poultry or lambs, at least among reintroduced animals, but not significantly in terms of numbers [20].

Lynx territory sizes of around 5 km^2^ are described by ecological studies for resident female lynxes [18]. Such values were mentioned by 14% of key actors, but most respondents to this question answered that they either did not know or mentioned a wider occupancy (54%). What actors considered as territory was different from scientific literature. Territory was interpreted by most interviewees not just as an individual stable range as in Ecology but as all the space used by animals including dispersal movements of individuals. The dispersion capacity of some lynxes, namely from Spain to Portugal or vice versa, had been recently popularized by the media, so long distance journeys were integrated in interviewees’ knowledge about lynx ecology.

With respect to the superpredator effect of the lynx over other carnivores, only 25% of interviewees in all areas were sure that lynx could remove foxes and mongooses from an area. This lynx behavior was observed by ecological studies [19] and has been publicized by conservationists. A few older informants, who had been in contact with the lynx in the past also knew about it:

In the areas where the lynx patrols, they (the other carnivores) don’t appear much, because they are afraid of the lynx, the lynx is a strong animal, very strong, and that is why these other animals are not very frequent (…) It is said that he will come back, let’s see. I would very much like to see it around here, it is a very pretty animal (...). (Lynx observer, MB 2013)

Other key actors considered scientific knowledge imposed and produced elsewhere and not necessarily valid in their region. The community rather values direct experience as a form of acquiring empirical knowledge. The actors mentioned a discrepancy between local knowledge and decisions based on knowledge “from the office” (*n* = 14), and in fact, we registered some reaction towards the dominance of scientific literature over local knowledge and everyday evidence about wildlife:

(The Natural Park)… when they come to me with their teachings…they want to teach us! They only find wild animals here because we have preserved them! (Manager, G 2014)

When we analyzed knowledge by key actor’s profile, we observed that technicians and observers of lynx tended to identify the lynx as a rabbit specialist. Technicians also tended to give precise territory estimation and be certain about the superpredator effect of lynx. Council representatives, managers, and land owners more frequently answered “I do not know” to the diet question. There were no significant differences in the biological knowledge about the lynx between hunters and non-hunting key actors.

Chi-square and Spearman’s correlation indicated a significant positive correlation between knowledge of lynx diet and positive opinions about reintroduction. The closer the knowledge about diet was to scientific literature, the more favorable an actor could be to the possibility of lynx living in the area (*ρ* = 0.264; *n* = 110; *p* = 0.005). The other variables of knowledge––about territory size and superpredator effect––did not show a robust association with opinion. These results, different for the three variables, indicate that possessing knowledge similar to scientific knowledge will not necessarily influence acceptance of reintroduction.

Concerning differences among geographical areas, we only found significant differences with respect to knowledge about the lynx superpredator effect. In Malcata, 44% of key actors do not believe lynxes have an effect on other species. It was in Guadiana that 57% of the interviewees referred to this effect of lynx over other carnivores. This particular knowledge in Guadiana seems to have been partly a result of recent contact with scientific biological expertise and local conservation projects.

### Advantages and disadvantages of reintroduction

**Fig. 3:**
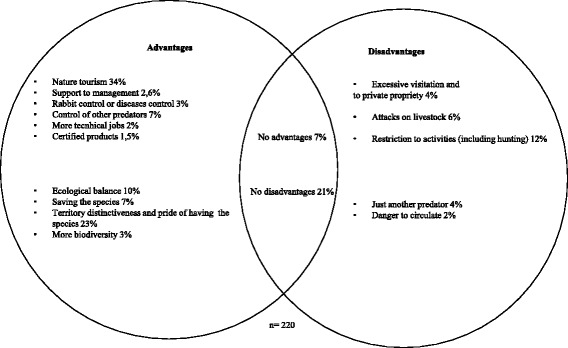
Advantages and disadvantages of lynx reintroduction freely named by key actors. Frequency of occurrence and total number of answers indicated

Respondents freely named more advantages than disadvantages when evaluating the possibility of reintroduction (Fig. 3). The most frequent advantages of the lynx’ future presence were nature tourism and territorial distinctiveness. As birdwatching and other nature tourism activities have been steadily growing, particularly in Guadiana, actors have expectations associated with tourism. Hence, locals also considered an advantage of “having” a rare and emblematic species that does not exist anywhere else.

Among a total of ten advantage categories identified by actors, five followed ecological criteria, namely prey disease control, control of other predators, more biodiversity in the area, ecological balance in the ecosystem, and saving the species (*n* = 32). The last two were mentioned by a total of 19 actors just in Guadiana.

Among disadvantages, the restriction of activities, namely of hunting practices, again, was the main negative association with lynx presence (12%). Potential attacks on livestock, a common source of conflict between humans and predators, were mentioned less (6%), and mainly in Guadiana. Disinterest or opposition to lynx presence with the opinion “no advantages” occurred mostly in this area also. Interestingly, the disadvantage “excessive visitation” in Guadiana partly reflects the concerns of private owners and discords with the potential expansion of nature tourism. There are different land use interests in the area which might be difficult to reconcile: tourism, livestock production, forestry, hunting, pedestrian access, or radical sports. In Guadiana, the existence of large fenced estates and restricted access to the public was a social issue raised in interviews.

In terms of different key actors, there was a tendency for hunting managers to mention “no advantages” and for hunting guards not to mention “nature tourism.” Concerning the advantage “ecological balance” associated with the lynx, significant differences were found between hunters and non-hunters, with hunters less likely to mention it (*p* < 0.001).

### Loving the lynx

During the interviews, we noted an emotional involvement with the lynx expressed by personal descriptions of the species, interest in encountering a lynx, and occasional association of lynx with an emblem. We consider the emotional responses of actors towards the species as a personal experience that can influence perceptions.

Concerning the question about the possibility of observing a lynx in the wild, the majority of the informants in the three areas expressed positivity (69% in all areas together) and lynx observers valued the sightings. Some key actors showed indifference concerning seeing or not seeing a lynx.

(Guadiana, 16%; Moura-Barrancos, 4%). Only one informant in Malcata (3%) said he did not want to see a lynx. There were no significant differences among geographical areas.

Spontaneous descriptions of the lynx also demonstrated appreciation. The dominant adjectives used by 46% of interviewees in Moura Barrancos and Guadiana referred to esthetic values such as “beautiful” and “pretty.” Furthermore, 42% expressed some kind of admiration or fascination: “strong,” “admirable,” “elegant,” “spectacular,” “nice,” “astute,” “majestic,” “interesting,” “important,” and “fantastic.” Only 5% of the lynx descriptions contained negative adjectives using the terms “terrible,” “beast,” or “predator,” for example:

“It looks like a robust, strong animal. It is a predator, it looks terrible (...) the way they hunt is probably terrible.” (Hunting manager, G 2014)*.*

Fear of lynxes does not seem to be an important issue in the three geographic areas. Five informants (four of which from Guadiana) mentioned fear of the lynx and considered it a dangerous animal to humans, while eighteen people affirmed that the lynx was harmless to humans, in Guadiana and in Moura-Barrancos.

Interviewees from Moura-Barrancos and Malcata further expressed that the lynx was a species that historically belonged to their area, thereby demonstrating a sense of pride. Lynx was further described as an emblem, a symbol for an area because of its charisma and beauty (an aspect also developed in [57]).

“It could be a symbol, a reason, an emblem, an excuse to build a sanctuary in the hills for everybody.” (Nature activity user, MB 2013) 

From what we could statistically explore, aesthetical appreciations of the lynx were not significantly related either to positive opinions on reintroduction or to the positive will to see a lynx in the wild.

### Local perceptions of conservation initiatives

**Fig. 4:**
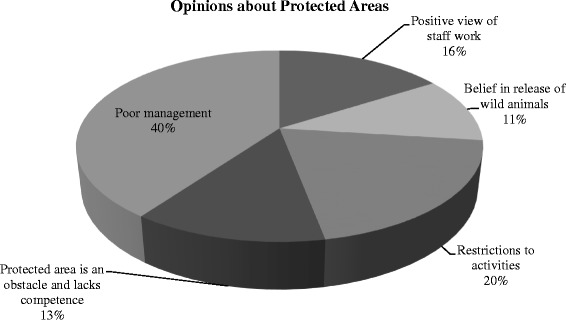
Key actors’ impressions about protected areas. Percentages refer to occurrence of each opinion. Each key actor could express more than one opinion

**Fig. 5:**
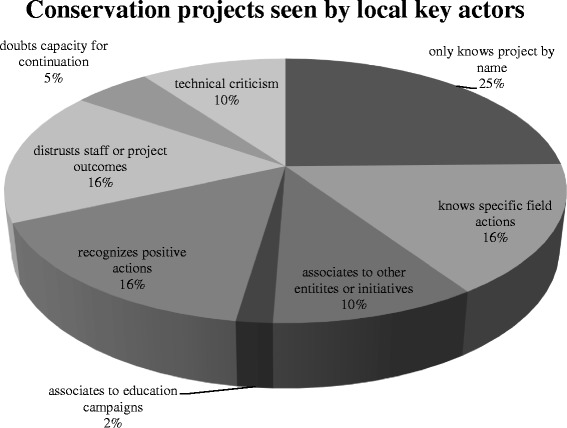
Key actors’ impressions about conservation projects in their areas. Percentages refer to occurrence of each opinion. Each key actor could express more than one impression

**Fig. 6:**
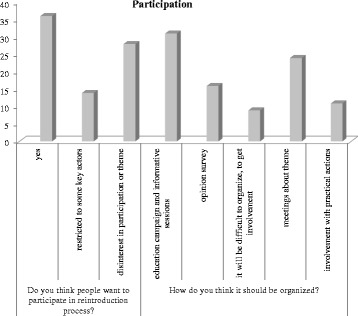
Opinions about local participation in the process of reintroduction

Figures 4, 5, and 6 summarize key actors’ impressions about conservation projects, the management of protected areas and their willingness to participate in reintroduction as a local conservation process.

In general, although positive views were presented (16%) a critical view of conservation management was put forward (Fig. 4). Discourses revealed resistance to some bureaucratic procedures and administrative obstacles that a protected area represents to human activities. Actors also showed discontentment regarding potential restrictions in land use due to protection area classification. Additionally, key actors in Guadiana and Moura-Barrancos alluded to the state’s incapacity to keep long-term compromises and to attend to local peoples’ interests (*n* = 13). There was a recurrent belief, in particular, in the reintroduction area, that the administration releases wild animals (*n* = 16).

In the same tone, independent conservation projects were seen by locals as distant. Lack of information or involvement was emphasized together with distrust (Fig. 5). Recognition of concrete actions in terrain and positive outcomes was much less frequent among opinions. We believe this affects attitudes towards lynx reintroduction although a direct and statistically significant relationship in our data could not be found.

When asked about participation in the reintroduction decision or other conservation processes, key actors opinions could be divided into: (a) agreeing with participation, (b) considering the opinion survey a type of participation, (c) restricting the participation to certain key actors, or (d) referring that it is generally difficult to get local people to be interested and involved in most local issues (Fig. 6). Overall, more information and meetings about this process in particular were demanded by key actors.

## Discussion

Assuming an ethnoecological perspective, we characterized a reintroduction context in which (a) conflicts between local populations and central political decisions were revealed, (b) differing perceptions about wildlife and nature conservation were exposed, (c) local ecological knowledge has been in contact with scientific biological expertise, and (d) there is expectancy in a new scenario for human and non-human interactions.

### Acceptance and social positioning

In this study, we aimed to understand the attitudes of local key actors towards the lynx and towards the process of its reintroduction. Our quantitative results show variable levels of support among study areas––from 52 to 89%––and, in general, lower levels of support than other similar assessments. In fact, in Andalusia, 90% agreement with Iberian lynx reintroduction was found in a telephone survey [37], while a wider online survey also reported 90% public support towards Eurasian lynx reintroduction in the UK [36]. Caruso and Perez [35] found 95% support for the return of jaguars to an Argentinian province, independently of respondents’ gender, age, or location. The main difference of our survey to these studies is methodological. Studies conducted with an exclusive biological focus can suffer from using biased questions that can strongly influence results. Their analysis is also often restricted to a quantitative approach while we used open coding to analyze interviews and consider the whole diversity of responses. Furthermore, large numbers of inquiries among people who do not have to coexist with predators in their lands tend to enhance favorable opinions to wildlife presence. Lower percentages of support from key actors and mixed views about a predator can be expected with a local and non-random approach (see also Bowen-Jones [51]). Castro et al. [38], surveying local opinion about the protection of the lynx in the Portuguese southern areas, found 58% of residents supporting it, a figure closer to our data in the reintroduction area. Similar to our data, that study also found considerable ambivalence among residents interviewed randomly. We attributed the higher ambivalence in Guadiana as a reaction to the announcement of the decision to reintroduce in 2014 as the survey was taking place.

Firstly, at the time of the study, the Council of Mértola (the main town in the reintroduction area) held an ambiguous position, and later, there was public opposition. Negotiations between administration and land owners were ongoing, and a general atmosphere of impasse was created. Furthermore, Malcata and Moura-Barrancos were areas of historical presence of lynx where the species was previously known for a long time. People inhabiting such areas tend to be more tolerant of carnivores compared to people experiencing carnivore reintroduction (e.g., [28, 52]).

### The role of knowledge

Our data stresses how local knowledge about a species may be heterogeneous in local communities, being constructed by some from direct experience, and for others also as the result of encounters with conservation professionals. Local ecological knowledge in this case study has a hybrid character which was described in other Portuguese rural contexts [53]. Some authors associate knowledge of carnivore biology to favorable attitudes towards conservation, namely Bath et al. [33], who found a positive correlation between attitude score and knowledge about the Eurasian Lynx. We did find, however, that a certain discrepancy with scientific knowledge about the lynx does not emerge as a predictor of opposition towards reintroduction. The association between opinions about conservation and the role of knowledge is not compulsory. Other authors like Johns [54] have confirmed that, and Ericsson and Heberlein [30] found that hunters in areas with wolves had the most accurate knowledge about them but at the same time, the most negative attitudes. Attitude towards predators’ presence may depend mainly on the specificities of each social context and the particular animal species rather than knowledge. This conclusion fits with other ethnographic surveys in a European context that showed the different impact of three carnivore species on human perceptions [55] and the importance of the socio-economic context [56]. Dressel et al. [57] also emphasized factors affecting attitudes towards large carnivores such as the animal’s presence, changes in policy and economics, and media coverage.

Studies on jaguar add that attitudes, tolerance, and social norms vary across and within communities [58]. Accordingly, our study indicates that each context has to be studied in detail and variables that condition attitudes in one case might not in another.

### Which contestation?

Our qualitative analysis allowed a description of local contestation revealing reasons for some resistance to lynx reintroduction in Portuguese territories and revealing strengths of the process from an *emic* perspective. Most local voices stated that lynx reintroduction in their lands was conditional upon a prior increase of wild rabbit abundance and a financial return. These claims are linked to the hunters’ sense of ownership over the lynx’ main prey. Hunter organizations publicly allude to having invested in increasing rabbit density and therefore see lynx as a competitor rather than an ecological balancer (a complementary analysis of our data also details this issue in [59]). Due to a recent decline in wild rabbit numbers, the hunting business, particularly in the reintroduction area, is under economic constraints. In fact, hunters’ voices differentiate themselves in being significantly associated with the claim of financial compensation and in presenting a more dominionistic orientation towards wildlife [59]. On the other hand, being a hunter was not a factor in holding a negative opinion towards reintroduction itself. So, hunters contested lynx reintroduction but were not necessarily against the species’ presence.

The focus of the local narrative on financial compensation accompanies the global experience of materialism, predator conflict compensation and the wider process of objectification and commodification of nature itself [60–62]. Nature becomes mercantilized, and the discourse of certain local groups was centered on benefits. The return of a wild species announced by the administration, as our results show, is an opportunity for negotiation. It has been pointed out that in the narrative produced for the management of wildlife, namely on sustainable hunting, there are messages of purposive management of nature and dependence upon global capitalism [63]. The impact of a capitalist global perspective in rural discourses was partly expected in a western European context where landscape and lifestyle are in rapid reconfiguration. Our study areas were characterized by a history of social inequalities and, generally, no access to education or land ownership. Nowadays, there is still a low expenditure capacity per capita but agriculture is strongly ruled by a policy of subsidies designed within a European framework. Local communities, involved with the market economy since historical times, fear nature conservation as an obstacle to economic growth and wealth. Lynx reintroduction has been not only just a nature protection theme but also a negotiation process. Our case study describes a situation distinct from others where indigenous groups have been seen as nature protectors or “ecologically noble savages” [64]. Both local discourses and administration follow logics of commodifying wild species, considering them as natural resources to exploit. Our local communities and administration seem to have different and conflicting positions but those are not, in fact, based on opposite versions of nature as for instance Aiyadurai ([65]: 313] describes for the case of the tiger in India.

Part of our key actors’ enumeration of the advantages of lynx reintroduction further expresses a material expectancy. Local actors see the exploitation of the lynx as a possible tourist attraction and its threat status as a high visibility factor: “From the touristic point of view the lynx has an incalculable value, if it is worked out. It can have a commercial value. It is already the most threatened felid in the world. I am in favour of its conservation.” (Guadiana 2014)*.* The conciliation of conservation and economic gains is a common perspective among administrations and conservationists (e.g. [66]), and it does not necessarily hinder the return of the lynx. However, concerning this aspect, authors such as McCauley [67] alert that market-based conservation strategies might not always be a solution to protect nature and there might be an illusory aspect to this discourse. This is particularly important when there is conflict with human interests. Predators can generate conflict and indeed even in cases with widespread social support, such as jaguar reintroduction in Argentina, attitudes can change after the species impacts the economy [35] in rural areas. Accordingly, we foresee that the general favorable trend in our areas may decrease if local livestock losses attributed to the lynx occur, in particular among sheep farmers, a group which deserves further attention.

### A beautiful symbol, the moral agreement

The second main finding of our study is the positive relation of local actors with the species due particularly to aesthetical appreciation of the wild felid, identification with a potential territory emblem, and the existence of moral agreement with its protection. This was demonstrated by the high percentage of actors who wished to encounter a lynx together with the statement that saving the species was an advantage of reintroduction. There was a sense of pride in having a rare species that confers distinctiveness upon the territory. A wild species historically considered as a vermin to be exterminated is presently a global symbol of conservation that has been locally appropriated [21]. Furthermore, our key actors considered the species harmless. In places where humans coexisted with large predators, fear has been a predictor of negative attitudes [33] and conflicts are often described [68]. Ainsworth et al. [69] mention how social values such as emotional attachment can make a difference regarding support for the conservation of a particular species. Among our results on the advantages of lynx reintroduction, we also highlight local voices in favor of “saving the species,” which echo the “deep ecology” movement or “restoring nature” philosophies [70]. This discourse has implicit a moral obligation of humanity to protect nature or the idea of Naess “humans have no right to reduce richness and diversity of life” [71]. These are important non-economic aspects of how reintroduction of a wild species is seen by locals. Such factors also seem to have shaped perceptions about wild felids such as the lion in Africa [39]. Multiple facets of discourses introduce diversity into the argument about which benefits and disadvantages conservation and wild species bring to local populations and to ongoing negotiations. As Milton ([72]: 108) summarizes, “emotions generate feelings which motivate action” and a “recognition of the fundamentally emotional character of all personal commitments is essential if we are to understand any public discourse, including that on nature protection.”

### Living in protected areas

The considerable criticism and reference to restrictions by local actors concerning protected areas shows the experience of an imposed model of nature conservation. Although protected areas are not just seen negatively, their existence was mostly a response to environmentally normative and external international pressures. This is still an underlying and unresolved human-human dimension [73] of our local scenarios rather than a conflict with a specific predator which is often publicized.

Accordingly, the administration is believed to own and release wild animals such as foxes. This perception was noted elsewhere (authors’ personal data and [74]) and has a social significance. We propose that this rumor (as foxes or other carnivores were not actually reintroduced in Portugal) is a response to the recent management of the territory within a biological framework. It could also have been founded on the observation by locals of particular administration practices: (1) In the 1970s–80s, the Forestry Services took hunting restocking procedures (with Red deer) using discreet operations that sometimes took place during the night without public announcement (ICNF data); (2) mostly in the last decade, rehabilitated raptors have been released in protected areas; (3) wildlife monitoring occurs regularly in terrain implying circulation of vehicles and equipment. The technicians conducting such operations and the new biologists in the field relate to wild animals in an analytical way, a different type of relationship with wildlife that may have triggered the idea of the release of wild species. Not only does it seem possible from a local perspective but it also looks like a coherent way of protecting wildlife and managing protected areas. As West et al. [75] mention, there is a mismatch in the way nature is perceived and utilized.

The management of a territory for wildlife preservation in a non-participatory model of governance has likewise engendered some resistance among locals as our results about opinions on protected areas show. The creation of natural parks and reserves is associated with an original impulse from the nineteenth century of creating untouched areas where people have no place. In our present case, it is seen as the production of a “space and place” where fragile species can be more important than human activities. This is an important societal conflict that for instance Pienaar et al. [76] report for the cougar, another case of wild felid recovery.

The connection of rural residents with the natural world is also changing. From residents’ narratives, the trend is one of less direct experience of wild animals and more familiarity through the media. Lynx and other predators can be as close or distant a reality as exotic places seen in TV nature documentaries. As Aiyadurai ([65]: 305) mentions, there are “changing notions of nature in the age of globalization.”

This reconfiguration of nature and the feeling of distance of important key actors to conservation projects are of relevance for conservationists. The recognition of all actors such as local council representatives and their social role is crucial.

The opinions of key actors concerning local participation in conservation were diffuse. Decision-making and governance institutions shape people’s motivations and abilities to participate [77]. As a result of the Portuguese history of a long dictatorship regime and accentuated class differences, our rural communities do not have much experience of governance. A reintroduction scenario can be an opportunity to engage local actors in conservation and make the return of an iconic species into a participation process. The way locals relate with wild species and nature conservation will determine the success of reintroductions and wildlife protection policy in general. Nature conservation decisions should consider locals’ points of view, and attitudes should continue to be studied.

## Conclusions

We found variable levels of support for lynx reintroduction between different areas suggesting that each social context has its own specificities. Discrepancy between local knowledge about a wild species and scientific literature does not necessarily associate with unfavorable opinions about conservation. Local ecological knowledge can be constructed from memory of coexistence as well as technical information. Collective learning between local actors and conservation professionals might therefore be more effective than top-down education campaigns.

The heterogeneity of attitudes we found is an indicator of a new rural scenario where emotional, symbolic, and moral dimensions might play a role in nature management. However, western rural communities are not the “noble savages” and nature protectors as are other traditional groups, and actors tend to claim for benefits in a situation of reintroduction. Among local voices, financial compensation was significantly associated to hunters, meaning that the relational process with the predator is very much of rivalry as during historical persecution. Understanding complexity and diverse interests in local communities are useful in not oversimplifying local positions towards predator conservation and in uncovering aspects of the so called “wildlife conflict.”

Our results concerning the distant experience of actors living in protected areas can contribute to a critical reflection on relational processes with environmental normative and can have implications for conservation initiatives. Encounters between technicians and the local population expose differing perceptions about wildlife and conservation and that social reality should be addressed. We recommend that professional conservation teams rethink their image among local populations and increase proximity with different types of key actors.

We believe that the ethnographic approach used and the methodological choice of open interviews of pre-selected key actors, and open-coding categories allowed a better understanding of local voices and perceptions in their multiple dimensions.

There are perceived barriers to effective collaboration between social scientists and conservation biologists. Interdisciplinary studies have been exceptional although both natural and social scientists agree that better collaboration would contribute to conservation success [78]. The results presented in this paper were integrated in an international conservation project, providing an opportunity for local voices to be taken into account. During the project and the process of reintroduction, we advanced recommendations for participation and communication. Therefore, this is an example of how anthropology can be applied to species conservation and how the discipline can be part of solutions to environmental issues. A reintroduction team should include social scientists actively working with biologists and other technicians. An in-depth study can provide a baseline assessment and address local contestation. Our case study yielded more than simplistic acceptance or resistance levels by providing details on key actors opinions and positions and can be applied in future reintroductions.

Combination of quantitative and qualitative data analysis together with information on the context gives insights into the complexity of contemporary rural systems where nature is part of human activities and different conceptual perspectives coexist.

## Appendix

### Main questions from personal interview

1. Name
2. Profile
3. How long do you live here?
4. Are you a hunter?
5. I would like you to tell me what main changes occurred in the habitats around here?
6. Which species do you know here? (presenting image cards)
7. Have you ever seen lynx? How was it? Have you heard that it existed here in the past?
8. I would like you to tell me what you know about the lynx. Do you have an idea about the diet, what the species eats? Do you know the size of a territory of a lynx or a couple? Do you think this species has influence over these species (carnivore cards)?
9. Would you like to see a lynx in the wild?
10. Do you think it could live in this region?
11. Where around here you think it would be adequate for reintroduction?
12. How do you think these processes of reintroduction happen, to release the animals?
13. Do you think there would be differences for people if lynxes live here?
14. Which ones? Which main advantages and disadvantages do you think reintroduction brings?
15. (If danger is spontaneously mentioned) Do you think lynx can be dangerous? What about the wolf?
16. Do you remember the process of creating the natural park (or the Nature 2000 site or the natural reserve depending in which geographic area)? What is your opinion about it?
17. Do you think people want to participate in the reintroduction process?
18. How do you think it should be organized?
19. Have you heard about lynx conservation projects ongoing? What is your impression of these projects?
20. What impression do you have of the Institute for Nature Conservation and Forests (protected areas administration)?
